# Comparison of the use of screening tools for evaluating cognitive impairment in patients with Parkinson's disease

**DOI:** 10.1590/s1980-5764-2016dn1004015

**Published:** 2016

**Authors:** Carlos Henrique Ferreira Camargo, Eduardo de Souza Tolentino, Augusto Bronzini, Marcelo de Araújo Ladeira, Ronilson Lima, Gustavo Leopold Schultz-Pereira, Marcelo Rezende Young-Blood

**Affiliations:** 1 MD, MSc, PhD. Head of Neurology Service, Hospital Universitário, Universidade Estadual de Ponta Grossa, PR, Brazil.; 2 Medicine Student. Neurology service, Hospital Universitário, Universidade Estadual de Ponta Grossa, PR, Brasil.; 3 MD. Resident of Neurology Service, Hospital Universitário, Universidade Estadual de Ponta Grossa, PR, Brasil.; 4 MD, MSc. Neurologist of Neurology Service, Hospital Universitário, Universidade Estadual de Ponta Grossa, PR, Brasil.

**Keywords:** Parkinson's disease, dementia, cognition

## Abstract

**Background:**

Screening tests have been used for cognitive deficits in Parkinson's disease (PD).

**Objective:**

This study compared the Montreal Cognitive Assessment (MoCA) test, the Mini-Mental State Examination (MMSE) and the clock drawing test for this purpose.

**Methods:**

A total of 50 patients with PD were selected, 41 (82%) were diagnosed with dementia by the criteria of the Movement Disorder Society. The test Scales for Outcomes in Parkinson's Disease-Cognition (SCOPA-Cog) was used as the gold standard in comparison with the screening tests.

**Results:**

The MoCA test (AUC=0.906) had a sensitivity of 87.80% and specificity of 88.89%. When the MMSE was associated with the clock drawing test (AUC=0.936), it had a specificity of 66.67% and sensitivity of up to 97.56%.

**Conclusion:**

The study suggests that the MoCA test can be a good screening test in PD. However, MMSE associated with the clock drawing test may be more effective than the MoCA test.

## Abstract (pt)

**Embasamento:**

Diversos testes têm sido utilizados como ferramenta de triagem para déficits cognitivos na doença de Parkinson (DP).

**Objetivo:**

Este estudo comparou os testes *Montreal Cognitive Assessment* (MoCA), Mini Exame do Estado Mental (MEEM) e teste do relógio para essa finalidade.

**Métodos:**

Foram selecionados 50 pacientes com DP, dos quais 41 (82 %) foram diagnosticados com demência pelos critérios da *Movement Disorder Society*. O teste *Scales for Outcomes in Parkinson's Disease-Cognition* (SCOPA-Cog) foi usado como padrão-ouro na comparação com os testes de triagem.

**Resultados:**

O teste MoCA (AUC=0,906) apresentou sensibilidade de 87,80% e especificidade de 88,89%. Quando o MEEM foi associado ao teste do relógio (AUC=0,936), apresentou especificidade de 66,67% e sensibilidade de até 97,56%.

**Conclusão:**

O estudo sugere que o teste MoCA pode ser um melhor teste de triagem na DP. Entretanto, o MEEM associado ao teste do relógio pode ser mais eficiente que o MoCA.

## INTRODUCTION

Parkinson's disease (PD) is a neurodegenerative disorder characterized by movement disorders and non-motor symptoms,^1^ associated with progressive depletion of dopaminergic nigrostriatal and mesocorticolimbic neurons,^2^ abnormal deposition of z-sinuclein in remaining cells and gliosis in specific areas of the nervous system.^1^ Non-dopaminergic pathways are also involved, including the serotoninergic and cholinergic neurons, in addition to the spinal cord and peripheral nervous system, correlating with the main non-motor symptoms of the disease.^3^ Unlike the original descriptions of James Parkinson - "the senses and the intellect remain unharmed" -, today it is known that the PD is commonly associated with various non-motor features,^4^ including dementia, depression, apathy, visual hallucinations, autonomic dysfunctions, changes in sleep-wakefulness and in pain perception.^3,5^

Dementia affects about 40%^3,6,7^ to 80%^4,8^ of individuals with PD during the course of the disease (Parkinson's disease dementia - PDD). It is believed that 3% to 4% of cases of dementia in the general population are related to PD, and the estimated prevalence of PDD in the general population aged over 65 years is 0.2 to 0.5%.^9^ PDD has an insidious onset and progressive evolution,^6^ being a major cause of the increase in the need for care at old people's homes, higher costs in healthcare and increased mortality.^8,10-12^ The cognitive domains affected in patients with PDD are: attention, memory, executive function, construction and apraxia, visuospatial function and language.^6^ When dementia becomes clinically significant, the average survival of patients drops to 5 years.^12^

Several tests and scales have been used as tools for early identification of cognitive deficits in PD. Among them are the MoCA test (Montreal Cognitive Assessment), the Mini-Mental State Examination (MMSE), the test Scales for Outcomes in Parkinson's Disease-Cognition (SCOPA-Cog) and clock drawing test. The screening tests - MMSE, MoCA and the clock drawing test - aim to track individuals with mild to severe cognitive deficits. MMSE is the most widely used instrument in the detection of dementia^13-16^, measuring multiple domains of cognition. Although not developed specifically for patients with PD, it is consistently used in studies that include these patients^13^ and is still recommended and used as a tool for screening dementia in patients with PD^16^; however, its accuracy in these patients has been questioned.^14-16^ MoCA is a brief global cognitive instrument that has been used to evaluate cognition in patients with PD.^14^ The SCOPA-Cog is a useful, short, reliable, sensitive, and valid instrument for assessing cognition in PD patients. Persons with PD typically exhibit impairment in complex attention, executive functions, information retrieval, procedural memory, visuoconstruction, verbal fluency, and speed of information processing. Most of these cognitive functions are specifically tested by the SCOPA-COG. It can define the presence of dementia in individuals with PD.^17,18^

In this context, the aim of this study was to compare screening tools for assessment of cognitive impairment in patients with PD.

## METHODS

Fifty (50) patients, selected according to the United Kingdom Parkinson's Disease Society Brain Bank Diagnostic Criteria for Parkinson's Disease,^19^ treated at the Neurology Service of the Hospital Universitário Regional dos Campos Gerais (HURCG) and at INOVARE Serviços de Saúde Ltda., who agreed to participate were included in the study. Patients that exhibited clinical conditions which precluded proper cognitive assessment and/or application of the proposed tests were excluded, such as:

[A] advanced clinical conditions of the disease and/or severe sensory deficits;
[B] the presence of psychotic symptoms;
[C] the presence of another dementia other than that associated with PD.

The study was approved by the Research Ethics Committee (COEP) of the Universidade Estadual de Ponta Grossa (reference n# 631.285 FA).

All patients were assessed during the ON phase of Levodopa therapy, preferably two hours after the medication had been administered. The clinical assessment was carried out by a team trained in movement disorders. A semi-structured questionnaire was applied to collect epidemiologic data and data about disease progression and previous/current treatment. Patients were classified according to motor changes on the Hoehn and Yahr scale^20^ and the Unified Parkinson's Disease Rating Scale III^21^ (UPDRS-III).

Cognition was assessed using the test SCOPA-Cog, which consisted of 10 items. Verbal and non-verbal memory and learning are assessed by means of a cube test (in which the patient copies the order in which four cubes are pointed to) and by reading/recalling ten words. Attention is assessed by the patient saying the months of the year in reverse order and performing three serial subtractions. Aspects of executive functions measured include complex motor planning, working memory and verbal fluency. A figure assembly task to evaluate visuospatial function (patients are asked to determine which shapes are needed to construct another figure) is the last subtest in the scale.^13^ There is a maximum of 43 points , and a value lower than 22 points defined the cut-off for dementia.^17^ Dementia clinical signs were confirmed using the criteria of the Movement Disorder Society (MDS) for PDD.^21^ Based on results, patients were divided into a group of patients without dementia and another group of patients with dementia.

The screening tests applied were:

1) MoCA: a test scoring a maximum of 30 points which evaluates cognitive and executive functions. It features 6 questions for guidance and 5 words for a test of memory, in addition to assessing visuospatial function through clock drawing. The executive functions were evaluated using a reduced version of the trail test B, phonemic fluency and a task of verbal abstraction.^13^ A point was added in cases of subjects having 12 years or less of education.^16,22,23^
2) MMSE: is a test scoring a maximum 30 points, divided into: orientation (10 points), memorization and immediate recall (6 points), attention and concentration (5 points), oral and written language (8 points) and visuospatial function (1 point)^13,24^.
3) Clock drawing test: this consists of asking a patient to draw a clock with hands showing a specific time, scored on a scale from 0 to 10. Zero indicates absolute inability to draw and 10 for production of the entire drawing, with the hands marking the right, mutually agreed time, with the numbers well organized and distributed around the circumference^22,25^.

The groups were tested for normality using the Shapiro-Wilk test. All groups had a normal distribution. Statistical differences in means between groups were determined using the Chi-squared test or one-sided Student's t-test. Pearson's correlation coefficients were used to determine correlations. Fisher's exact test was used to determine differences between found and expected values. Results were expressed as mean±SD (standard deviation). ROC curves were built and values expressed in percentages for sensitivity, specificity, positive predictive value and negative predictive value. The values for AUC are given followed by the 95% confidence interval (CI) [AUC (95%)]. Differences were considered significant when p<0.05. The statistical analysis was performed with the software Statistics for Windows release 99 and Med Calc.

## RESULTS

Among the 50 patients diagnosed with PD, (male:female ratio was 1.88:1), 41 were diagnosed with dementia (82%) and 9 without dementia (18%). There was no significant difference between these groups in relation to age, age at onset of symptoms, disease duration, time in use of L-DOPA, or motor symptoms on both the Hoehn and Yahr and UPDRS-III scales. However, there was a difference between the groups in cognitive SCOPA-Cog and educational level (Table 1).

**Table 1:** Clinical and epidemiological characteristics of Parkinson’s disease patients with and without dementia.

Variables	Total (n=50)	With dementia Scopa-Cog <22 (n=41)	Without dementia Scopa-Cog ≥22 (n=9)	p
Gender	50 (100%)	41 (82%)	9 (18%)	1
Female	18 (36%)	15 (36.59%)	3 (33.33%)	
Male	32 (64%)	26 (63.41%)	6 (66.67%)	
Age	69.28±11.41	69.78±10.88	66.89±14.21	0.5055
Age at onset of symptoms	60.30±12.26	61.43±12.33	58.87±13.76	0.8431
Disease duration	8.22±5.25	8.45±8.77	6.89±5.71	0.6206
Time in use of L-DOPA	5.52±5.12	5.26±4.96	6.67±5.81	0.4326
Hoehn and Yahr	2.27±1.22	2.25±1.25	2.50±1.09	0.584
UPDRS - III	20.78±11.27	21.75±11.28	15.89±10.86	0.1618
Scopa-Cog	13.80±7.59	11.25±6.16	24.33±3.28	<0.001*
Educational level	6.90±5.58	5.925±4.99	19.44±6.38	0.01

* Age, educational level, disease duration, age at disease onset and time in use of L-DOPA expressed in years. UPDRS-III: Unified Parkinson’s Disease Rating Scale; Scopa-Cog: Scales for Outcomes in Parkinson’s Disease Cognition. *Statistically significant value.

**Figure 1:**
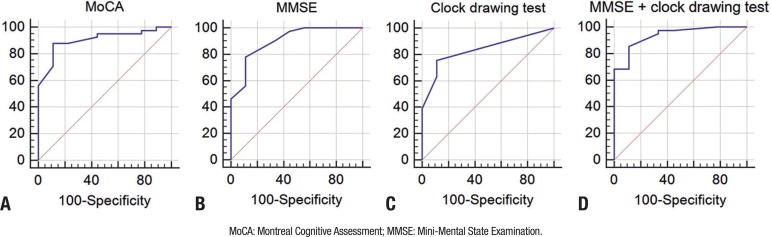
ROC curve for the screening test for cognitive changes in patients with Parkinson's disease.

On evaluation of the screening tests for cognitive deficits, relative to the SCOPA-Cog (gold standard), the MoCA test exhibited an AUC=0.906 (CI=0.788 to 0.970), p<0.0001(Figure 1A). Based on the ROC curve, the best cut-off score for detection of cognitive deficits in the studied sample was 19 points, with a sensitivity of 87.80%, specificity of 88.89%, positive predictive value of 97.29%, and negative predictive value of 61.52%. The parameters of the MoCA test were calculated with other cut-off scores (Table 2).

**Table 2:** Possible cut-off scores for cognitive deficits from ROC curve for MoCA and MMSE tests.

Test	Cut-off score	Sensitivity	Specificity
MOCA	≤19	87.80%	88.89%
	≤20	87.80%	77.78%
	≤21	92.68%	55.56%
	≤22	95.12%	55.56%
	≤23	95.12%	22.22%
	≤24	97.56%	22.22%
	≤25	97.56%	11.11%
MMSE	≤22	46.34%	100%
	≤23	56.10%	88.89%
	≤25	78.05%	88.89%
	≤26	90.24%	66.67%
	≤27	97.56%	55.56%

MoCA: Montreal Cognitive Assessment; MMSE: Mini-Mental State Examination.

The MMSE test had an AUC=0.902 (CI=0.785 to 0.968) p<0.0001, and with a cut-off of ≤26 points had sensitivity of 90.24% and specificity of 66.67%, positive predictive value of 47.06%, and negative predictive value of 96.97% (Figure 1B). The parameters of the MMSE test were calculated with other cut-off scores (Table 2). The clock drawing test had an AUC=0.837 (CI=0.785 to 0.926) p<0.0001, and with a cut-off of ≤8 points had sensitivity of 87.80% and specificity of 44.44%, positive predictive value of 96.87%, and negative predictive value of 44.44% (Figure 1C).

On the assessment of the MMSE in association with the clock drawing test there was an increase in AUC=0.936 (CI=0.829 to 0.986), p<0.0001(Figure 1D). For the same cut-off scores used in isolation with the clock drawing test (≤8 points) and MMSE (≤26 and ≤27), sensitivity was 95.12% (26 points) and 97.56% (=27 points) while specificity was 66.67% for both cut-off scores.

Examination of the domains of the MoCA test separately revealed a statistically significant difference between patients with and without dementia for all domains except naming (Table 3). Analysis of the domains of the MMSE test separately revealed a statistically significant difference between patients with and without dementia in the areas of spatial orientation, words to the contrary and recall of 3 words. There were also statistically significant differences between the groups of patients on drawing the clock test (Table 4).

**Table 3:** Domains of MoCa test in Parkinson’s disease patients with and without dementia.

	Patients with Parkinson’s disease	Patients with dementia (Scopa-Cog <22)	Patients without dementia (Scopa-Cog ≥22)	p
MoCA	16.06±5.92	14.66±5.43	22.44±3.36	<0.001*
Visuospatial/Executive	1.76±1.13	1.49±1.00	3±0.87	0.0001*
Nomination	2.34±0.82	2.24±0.86	2.78±0.44	0.0779
Attention	3.06±1.83	2.68±1.69	4.78±1.48	0.0013*
Language	1.58±1.07	1.44±1.03	2.22±1.09	0.0457*
Abstraction	1.06±0.82	0.93±0.82	1.67±0.5	0.0125*
Delayed Recall	0.78±1.18	0.54±0.84	1.89±1.83	0.0013*
Guidance	4.8±1.59	4.59±1.67	5.78±0.44	0.0404*

MoCA: Montreal Cognitive Assessment.

* Statistically significant value.

**Table 4:** Domains of MMSE test in Parkinson’s disease patients with and without dementia

	Patients with Parkinson’s disease	Patients with dementia (Scopa-Cog <22)	Patients without dementia (Scopa-Cog ≥22)	p
MMSE	22.96±4.75	21.98±4.6	27.44±3.28	0.0011*
Spatial orientation	3.9±1.44	3.70±1.52	4.77±0.44	0.0215*
Time orientation	4.4±0.92	4.31±0.98	4.77±0.44	0.089
Memorization of 3 words	2.94±0.42	2.92±0.46	3±0	0.322
Word to the contrary	2.84±1.96	2.46±1.92	4.55±1.01	0.001*
Recall of 3 words	1.44±1.1	1.26±1.11	2.22±1.09	0.012*
Language	7.1±1.1	7±1.16	7.55±0.72	0.088
Copy of Pentagons	0.34±0.47	0.29±0.46	0.55±0.52	0.068
Clock-drawing test	5.12±2.83	4.48±2.65	8±1.58	0.0002

MMSE: Mini-Mental State Examination.

* Statistically significant value.

## DISCUSSION

The results of this study showed significant differences in the comparison between the screening tests - MoCA, MMSE and clock drawing test - for cognitive deficits in PD patients. The use of an easy, fast and sensitive test for screening these deficits helps guide the decision-making process and referral for more specific neuropsychological testing.^16,28^ Therefore, the early recognition of cognitive deficits becomes important to allow selection of the appropriate treatment, thereby reducing disability and morbidity in these patients.^3,11,16,28^

In relation to the MoCA test, the value providing best balance between sensitivity and specificity occurred for the cut-off score of ≤19 points (87.80% and 88.89%, respectively). However, as it is sought for a screening test, a cut-off score with a higher sensitivity without greatly affecting specificity would be more useful. Thus, a value of ≤22 with a sensitivity of 95.12% and specificity of 55.56% would be adequate for the test purpose. Sobreira et al.^14^ determined a value of 21 points for determining cognitive changes on the MoCA test in PD patients, associated with sensitivity of 94% and specificity of 68%. In the present study, the values of sensitivity and specificity for 21 points proved similar to those found for 22 points. Thus, a value of 21 or 22 may be the ideal cut-off point for dementia on the MoCA test.^14,29^ Another study in patients with PD that suggested higher values - 24/25 points - had sensitivity of 82% and specificity of 75% in screening dementia.^16^ When these cut-off scores were evaluated in the present study, sensitivity was 97.56% for both scores, but specificity was 22.22% (24 points) and 11.11% (25 points), respectively.

Four studies^16,21,30,31^ which used the MoCA test in populations with PD suggested that it can be particularly sensitive for mild cognitive decline observed in PD. The MoCA test proved to be a quick, easy-to-administer test, having good "test-retest" and inter-examiner reliability, in addition to covering a range of areas of cognitive domains and being sensitive for detecting executive dysfunction in patients with PD,^13^ Studies showed that the MoCA test can be better than the MMSE for assessing cognitive deficits in patients with PD.^14,29^

However, the present study showed that the MMSE is also a useful test for screening cognitive changes in PD patients. Both the value of 26 points (sensitivity of 90.24% and specificity of 66.67%) as well as 27 points (sensitivity of 97.56% and specificity of 55.56%) could be used as a threshold for cognitive changes in PD. These data are in agreement with Oliveira et al.^15^ who found a sensitivity of 94% and a specificity of 55% using 26 points on the MMSE for diagnosing cognitive changes in PD. In a study involving patients with PDD diagnosed based on the criteria of the fourth edition of the Diagnostic and Statistical Manual of Mental Disorders (DSM-IV), the MMSE had a sensitivity of 98% and specificity of 77%.^32^

According to Chou et al.,^13^ the MMSE has proved to be a test that has good "test-retest" and inter-examiner reliability in the general population. One of its advantages is the possibility of measuring cognitive deficits over time, especially in patients with dementia (loss of 2 to 2.5 points per year). Although it is an easily administered screening tool, it does not adequately evaluate cognitive functions of reasoning, planning and executive functions, which are areas commonly affected in PD.^5,13^ Moreover, the MMSE is believed to be relatively insensitive for mild cognitive deficits.^13,16,28,33^

In the present study, separate observation of the MoCA domains found that the visual-spatial/executive functions, attention, language, abstraction, delayed recall and guidance differed statistically when comparing the groups with and without dementia. Separate examination of the MMSE domains revealed statistically significant difference in the areas of spatial orientation, words to the contrary and recall of 3 words between patients with and without dementia. The patients with PD committed more mistakes on the MoCA test compared to the MMSE test in areas typically affected in PDD, such as attention, executive function and visual-spatial processing. This suggests that the MoCA test was more sensitive than the MMSE for screening early cognitive impairment in PD. The cognitive profile of patients with PDD includes deficiencies or deficits in attention, learning, memory and visual-spatial memory functions. The MoCA test covers a larger number of tasks and these are more complex for evaluating executive function skills, language, memory and visual-spatial processing. For example, the MoCA uses more words for learning and a wider range in the assessment of delayed recall.^28^

Akin to the present study, other authors have also stated that the MoCA test can provide an accurate picture of the cognitive state of patients with PD when compared to other simple screening tools such as the MMSE, suggesting that the MoCA would be a better choice than the MMSE, a tool widely used for cognitive screening.^14,16,28,29^ However, an important finding of this study was that use of the clock drawing test in association with the MMSE, led to an increase in the area under the curve and in sensitivity for the same cut-off values compared to use of the MMSE alone. The clock drawing test is a simple test that is widely used in the screening of cognitive decline in patients with Alzheimer disease. This test covers a range of areas of cognition such as verbal comprehension, memory, abstract thinking, planning, concentration and visual-constructive abilities. In addition, this test is considered a good test for screening cognitive deficits.^26^

Among the 50 patients diagnosed with PD assessed, 82% (41 individuals) had dementia and 18% (9 individuals) had no diagnosis of dementia. This high prevalence of cognitive alterations may have occurred due to the low number in the sample and also to the fact that the study was conducted at a reference center where patients arrive with some years of disease, increasing their chances of having dementia. The present study has further limitations, also reported by other authors, such as previous exposure to environments of formal testing, knowledge of specific items (for example, test items from the naming test) and other factors which may also limit the validity of the test when applied in different individuals and cultures.^13,14^

In addition, educational level is a major factor to consider when assessing cognition. In this study, the lower education in the PDD group could be considered a risk factor for dementia.^24^ However, the low educational levels observed in this sample, and especially such a large difference between groups, may have influenced the tests results. These patients may have had greater difficulty performing the cognitive tests.^22-25^ For the MoCA and the MMSE, education-corrected scores were used to minimize the influence of lower educational level.^13,16,22-24^ Nevertheless, for the CDT there was no possibility of correcting for education and given this test is known to be susceptible to the influence of the educational level of patients, it is possible that results were overestimated.^22,25^

Thus, given the high prevalence of dementia in patients with PD, routine screening is needed to detect early cognitive deficits in these patients. This study suggests that the MoCA test can be a better test for assessing cognitive functions in PD. However, when the MMSE is used in association with the clock drawing test, the results, such as screening for PDD, seem to suggest results that are as effective or more effective than the MoCA test. Regardless of the test chosen, this study demonstrates that there are simple, quick and easy-to-apply tests which can be useful for detecting changes that are limiting and increase mortality in PD.
